# Silver Camphor Imine Complexes: Novel Antibacterial Compounds from Old Medicines

**DOI:** 10.3390/antibiotics7030065

**Published:** 2018-07-26

**Authors:** Jorge H. Leitão, Silvia A. Sousa, Silvestre A. Leite, Maria Fernanda N. N. Carvalho

**Affiliations:** 1 IBB—Institute for Bioengineering and Biosciences, Department of Bioengineering, Instituto Superior Técnico, Universidade de Lisboa. Av Rovisco Pais, 1049-001 Lisboa, Portugal; sousasilvia@tecnico.ulisboa.pt (S.A.S.); silvestre.leite@tecnico.ulisboa.pt (S.A.L.); 2 Centro de Química Estrutural, Instituto Superior Técnico, Universidade de Lisboa. Av Rovisco Pais, 1049-001 Lisboa, Portugal

**Keywords:** silver, camphor derivatives, silver camphorimine complexes, antimicrobial activity

## Abstract

The emergence of bacterial resistance to available antimicrobials has prompted the search for novel antibacterial compounds to overcome this public health problem. Metal-based complexes have been much less explored than organic compounds as antimicrobials, leading to investigations of the antimicrobial properties of selected complexes in which silver may occupy the frontline due to its use as medicine since ancient times. Like silver, camphor has also long been used for medicinal purposes. However, in both cases, limited information exists concerning the mechanisms of their antimicrobial action. This work reviews the present knowledge of the antimicrobial properties of camphor-derived silver complexes, focusing on recent research on the synthesis and antimicrobial properties of complexes based on silver and camphor imines. Selected examples of the structure and antimicrobial activity relationships of ligands studied so far are presented, showing the potential of silver camphorimine complexes as novel antimicrobials.

## 1. Introduction

The discovery and use of antimicrobials is a landmark of modern medicine. Most of the antimicrobials currently in use were developed last century, between the 1940s and 1960s [1,2]. These “magic bullets” have since then saved millions of human lives. However, bacterial resistance to multiple antimicrobials has increased worldwide over the last decades, mainly due to their misuse and abuse. Antibiotic resistance poses a serious threat to infection treatment, and thus a significant pressure on health systems, and is considered a major menace to human health [3,4]. A particular group of bacteria, referred to as the ESKAPE group (comprising *Enterococcus faecium*, *Staphyloccus aureus*, *Klebsiella pneumoniae*, *Acinetobacter baumanni*, *Pseudomonas aeruginosa*, and *Enterobacter* species) has emerged worldwide, with an increasing prevalence in hospitals and resistance to antibiotics [5,6].

Despite this emergence of resistance, the number of new antimicrobials reaching the market has been declining since the 1990s [7]. Multiple factors, including low investment return and regulatory requirements, are the major reasons [8] for the exodus of pharmaceutical and biotechnological companies from antimicrobial research and development.

Due to the new and emergent risks posed by bacterial resistance, the World Health Organization (WHO, February 2017) [9] has appealed for the investment of publicly funded agencies and the private sector in the research and development of new antibiotics. Motivated by the urgent need for new and effectively active antimicrobials, we have initiated the investigation of the antimicrobial activity of silver camphorimine complexes. Silver and camphor are medicines empirically used since ancient times, although the mechanisms underlying their antimicrobial activities remain to be fully understood.

## 2. The Antimicrobial Properties of Silver 

**Figure 1:**
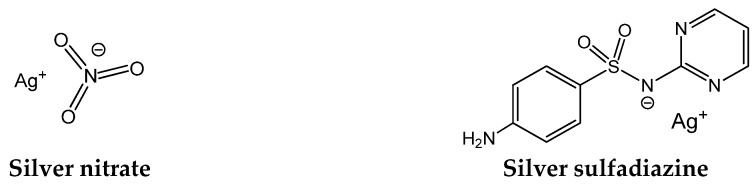
Examples of silver compounds with antibacterial properties.

Silver (Ag^0^) vessels were used in ancient times not only for aesthetic reasons but because they preserved the quality of water. Although microbes were unknown, the consequences of microbial contaminations were well known, and silver was a great help to control them. Aware of that, Hippocrates (the father of medicine, 5th to 4th centuries BC) prescribed silver preparations against infections. Later, the Romans extended the medicinal use to silver nitrate (AgNO_3_, Figure 1), and during the Medieval Period, it continued to be used for the treatment of skin wounds and ulcers [10,11]. Silver sulfadiazine (Figure 1) came later and is still in use to treat infections associated with burns [12,13].

Nowadays, silver metal and silver nanoparticles are used for medical device coatings and the reduction of bacterial adhesion to the surfaces of implants as gels or films [14,15]. Silver composites are used as antimicrobials against Gram-positive and/or Gram-negative bacteria [16,17,18]. Therapeutic and antiseptic applications of silver and silver derivatives actually extend to the control of surgical infections [19], a domain of increasing concern.

Despite the recognized antimicrobial activity of silver salts (e.g., silver nitrate, silver sulfadiazine) and silver particles, the pharmacological uses of silver derivatives have been restricted mostly to external uses in creams and dressings due to toxicity concerns. Recent studies showed that silver nitrate and silver nanoparticles have no significant toxicity, although AgNO_3_ accumulates more than silver nanoparticles in the organs of rats [20].

Notwithstanding years of use, only recently has some information been gathered on the molecular mechanisms underlying the antimicrobial properties of silver. Proteins have been pointed out as the major targets of silver ions, which can react with thiol groups, inactivating membrane-associated enzymes (e.g., those involved in electron transfer and energy generation) as the Na^+^-translocating NADH:ubiquinone oxidoreductase [21]. For instance, the addition of AgNO_3_ to *Escherichia coli* has been shown to lead to proton motive force collapse and subsequent cell death [22]. 

Using reflectance Fourier transform infrared (ATR-FTIR) spectroscopy, Ansari et al. [23] investigated changes in *E. coli* lipopolysaccharide (LPS) and l-α-phosphatidyl-ethanolamine (PE) upon exposure to silver nanoparticles. These authors showed that the LPS *O*-antigen was involved in the interaction with silver nanoparticles through hydrogen bonding. In addition, the silver nanoparticles induced the break of the phosphodiester bond of PE, forming phosphate monoesters and resulting in a highly disordered alkyl chain, most probably causing the destruction of the membrane and cell leaking.

Dibrov et al. [24] reported that low concentrations of silver ions induce massive proton leakage and loss of cell viability in *Vibrio cholerae*, suggesting that the antimicrobial activity of Ag^+^ results from its unspecific action on membrane proteins and/or the Ag^+^-modified phospholipid bilayer. Consistently, the increase in d/cis ratios of unsaturated membrane fatty acids were reported upon exposure to silver species [25], most probably affecting membrane fluidity and culminating with membrane integrity loss [23,26]. A proteomics study carried out with *Pseudomonas aeruginosa* revealed that treatment with silver nanoparticles led to the identification of 59 proteins related to membrane functions and intracellular oxygen reactive species generation, and 5 silver-binding proteins were found by proteomics [27]. Feng and colleagues [28] showed that, upon exposure to silver ions, *E. coli* and *S. aureus* lost the ability to replicate DNA, suggesting that the nucleoid is another bacterial structure targeted by silver ions. 

A few studies have also used electron microscopy techniques to inspect the effects of silver nanoparticles on bacterial cell morphology. An example is the work by Huang et al. [29], who used transmission electron microscopy (TEM) and scanning electron microscopy (SEM) to study the antimicrobial mechanisms of catechol-functional chitosan silver nanoparticles on the Gram-positive *S. aureus* and the Gram-negative *E. coli*. These authors suggest that the silver nanoparticles killed *S. aureus* through disruption of the cell wall and the consequent membrane damage and cytoplasmic content leak out. In the case of the Gram-negative *E. coli*, the antimicrobial mechanisms involved the adsorption of the silver nanoparticles to the surface of surface bacterial cells, interaction with the outer membrane, and damage of its permeability. This change in permeability was suggested to allow the silver ions to enter into the cytoplasm, interfering with cellular functioning.

Silver nanoparticles have also been reported as active against a wide range of multidrug-resistant (MDR) bacteria, including the Gram-positive *S. aureus*, *S. epidermidis*, *Streptococcus mutans*, *Enterococcus faecalis*, and *Bacillus subtilis*, and the Gram-negative *E. coli*, *V. cholerae*, *P. aeruginosa*, *Klebsiella pneumoniae*, and *Salmonella typhi* [30].

## 3. The Biological Properties of Camphor 

**Figure 2:**
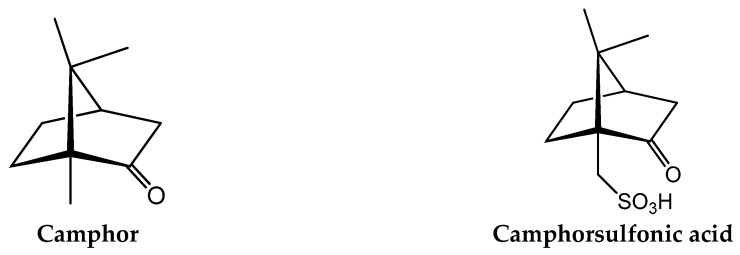
Molecular structures of camphor and camphorsulfonic acid.

(1*R*)-(+)-camphor (Figure 2) is a natural product (also produced by synthetic means) with very ancient applications as an insect repellent, muscular relaxant, and anesthetic [31,32] and is currently used as a cough suppressant and decongestant according to conditions established by the Food and Drug Administration. Recent studies show that camphor derivatives may also have relevant antimicrobial [33], antiviral [34,35], and/or cytotoxic properties [36], being also used as photoinitiators [37] or neuro-blocking agents [38]. However, in contrast with some knowledge of silver antibacterial mechanisms, such knowledge is inexistent in the case of camphor or camphor derivatives.

The wide range of pharmacological applications of camphor has fostered studies on the properties of camphor derivatives and camphor complexes. Camphor derivatives can be obtained by the introduction of suitable substituents on the camphor molecule (e.g., imine or carboxylic groups), keeping the camphor skeleton intact. Camphor imines, camphor carboxylates, camphor sulfonimines, and camphor sulfonamides (derived from camphorsulfonic acid, Figure 2) are among the camphor derivatives with suitable coordinating properties, some of which (e.g., camphor sulfonamides) display therapeutic properties [39].

Such as for silver and silver derivatives, not much information is available on the molecular mechanisms underlying the antimicrobial activity of camphor derivatives. Recent computer simulations of interactions with the viral surface hemagglutinin (HA) glycoprotein suggest that camphor derivatives inhibit HA activity by binding to hydrophobic sites of the protein [40]. Apparently, camphor imines are able to effectively block conformational rearrangements of HA, required for membrane fusion during virus entry in host cells. 

## 4. Silver Camphorimine Complexes

In complexes, the characteristics of the metal and the ligands combine in a cooperative way to generate compounds with distinct and frequently improved properties relative to the precursor species. Complexes have a wide variety of applications in catalysis, medicine, and the materials industry. Furthermore, properties of complexes can be tuned towards specific applications by varying the metal and ligands. The catalytic properties of silver complexes have been explored to promote organic transformations and bio-conjugation [41]. In the last decade, the evaluation of the biological properties of silver complexes has garnered considerable attention, in particular, fostering their use as antimicrobials or anticancer agents [1,4,42,43,44,45,46,47]. The therapeutic uses of silver complexes include silver sulfadiazine for treatment of infections associated with burns.

**Figure 3:**
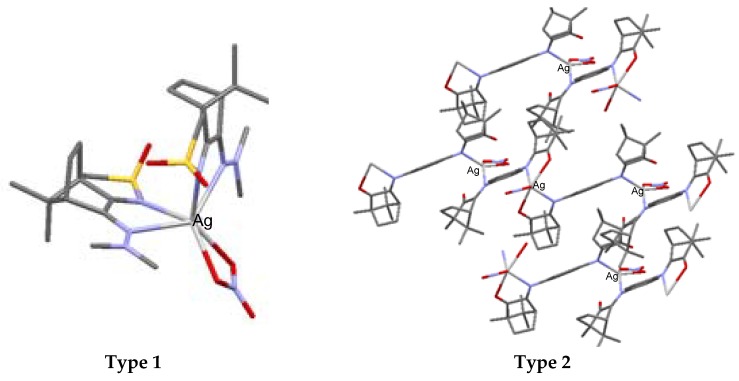
Structural arrangements corroborated by X-ray analysis in Ag(I)-camphor-derived complexes. The synthesis of the Ag(I) camphor complexes includes the preparation of suitable camphor compounds to be used as ligands, since just a few imine functionalized camphor derivatives are commercially available (e.g., camphor oxime and camphor sulphonylimine). (1*R*)-(+)-camphor is the starting material for the camphor compounds of type A1–A5 (Figure 4).

The core of the silver camphor imine complexes, the biological properties of which we have been studying, is formed by silver nitrate (AgNO_3_) that binds one or two camphor ligands per metal atom. Data obtained by X-rays show that nitrate acts as a bidentate ligand and that the camphor ligand binds the silver atom through the imine nitrogen atom. The complexes arrange according to monomeric (type 1, Figure 3) or polymeric (type 2, Figure 3) structures. The polymeric arrangement was typically found in imine and bicamphor imine complexes of general formula [Ag(NO_3_)L]_n_ [33], while the monomeric arrangement was found in camphor sulphonylimine ([Ag(NO_3_)L_2_]) [43] complexes (Figure 3).

(1*S*)-(+)-10-camphorsulfonic acid is the precursor for the tricyclic camphor sulfonylimine derivatives of types B1 and B2 (Figure 5) within a process that involves the sequential formation of chloride and amine derivatives prior to ring closure and oxidation to obtain 3-*oxo*-camphorsulfonylimine, which is then condensed with amines or hydrazines to afford camphor sulfonylimines of types B1 and B2 (Figure 5).

A wide variety of amines (YNH_2_) and hydrazines (NR_1_R_2_NNH_2_) can be used to tune the electronic and steric characteristics of the imine group at position 3 (see Figure 5 for labelling) of the camphor skeleton. Thus, the design and synthesis of camphor Ag(I) complexes can be driven to control electronic and steric parameters towards the aimed objectives. 

The camphor imine complexes, the antimicrobial properties of which were studied by us [42], were obtained in acetonitrile by the reaction of silver nitrate with the suitable camphor ligand (L) under metal-to-ligand ratios directed to mononuclear (Equation (1)) or polymeric complexes (Equation (2)). Strict control of the experimental conditions (exposure to light and reaction time) is necessary depending on the electronic characteristics of the camphor ligands to inhibit Ag(I) reduction, the formation of silver nanoparticles (AgNPs) (Equation (3)), and products of oxidation of the camphor derivatives (e.g., camphorquinone).

(1) $${\mathrm{AgNO}}_{3}+2\;L\;\to \;\left[\mathrm{Ag}(NO_{3})(L{)}_{2}\right]$$

(2) $${\mathrm{AgNO}}_{3}+L\;\to \;\left[\mathrm{Ag}(NO_{3})(L)\right]$$

(3) $${\mathrm{AgNO}}_{3}+2\;L\;\to \;\mathrm{AgNPs}+\mathrm{camphorquinone}+\mathrm{other}\;\mathrm{products}$$

Studies carried out so far evidence that all silver camphor imine complexes display biological activity that, according to the structural and electronic characteristics of the ligands, favors antibacterial, antifungal, and/or anticancer activity [33,34,35]. In some cases, antibacterial, antifungal, and cytotoxic activity combine in the same complex [42,44]. Such performance is highly relevant to cancer treatments because opportunistic fungi and bacteria might develop when body defenses diminish due to the use of anticancer drugs.

## 5. Antibacterial Activity 

To date, several silver camphor complexes have been synthesized and characterized, and their antibacterial activity towards Gram-positive (*S. aureus*) and Gram-negative bacteria (*E. coli*, *P. aeruginosa*, *Burkholderia contaminans*) has been screened using the Kirby–Bauer disk diffusion method and quantified based on the determination of minimal inhibitory concentrations (MIC) by microdilution assay standard methods [45]. *E. coli*, *S. aureus*, and *P. aeruginosa* strains were chosen because these species belong to the ESKAPE group. *B. contaminans* is a member of the *Burkholderia cepacia* complex (Bcc), a group of Gram-negative bacteria capable of causing life-threatening respiratory infections, of particular severity among cystic fibrosis patients [46,47,53,54]. Bcc bacteria are intrinsically resistant to multiple antimicrobials, rendering their eradication difficult to achieve [55]. Photographs in Figure 6 illustrate results from disk diffusion and MIC assays, obtained with complex **1** (see Table 1) for *E. coli* ATCC25922 and *S. aureus* Newman.

**Figure 4:**
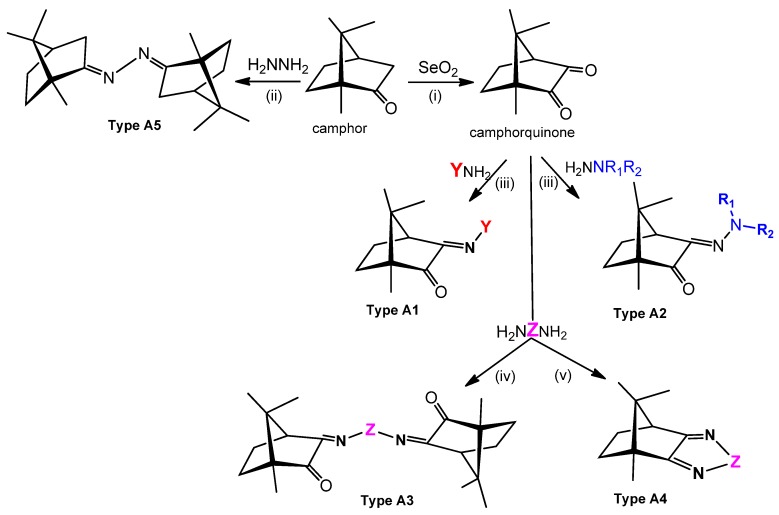
Camphor derivatives of the imine type: Y-aromatic or aliphatic; R_1_ = H, Me, Ph; R_2_ = H, Me, Ph, among others; Z = phenyl; biphenyl, linear alkane. For preparative details see references (i)—[48]; (ii)—[42]; (iii)—[49,50]; (iv)—[42,51]; (v)—[42,52].

**Figure 5:**
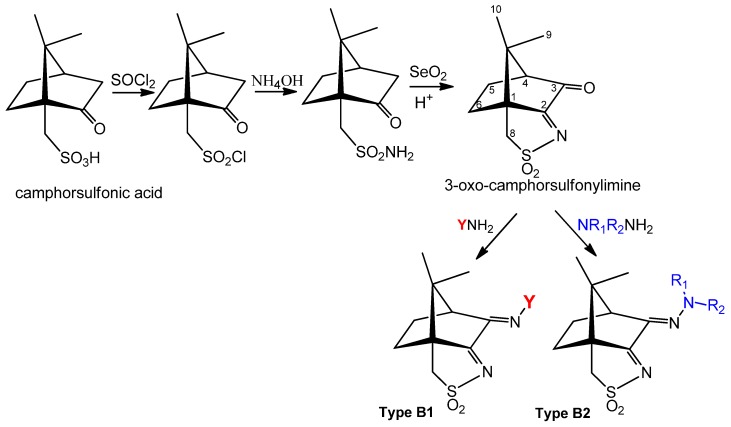
Camphorsulfonic acid derivatives of the camphorsulfonimine type.

**Figure 6:**
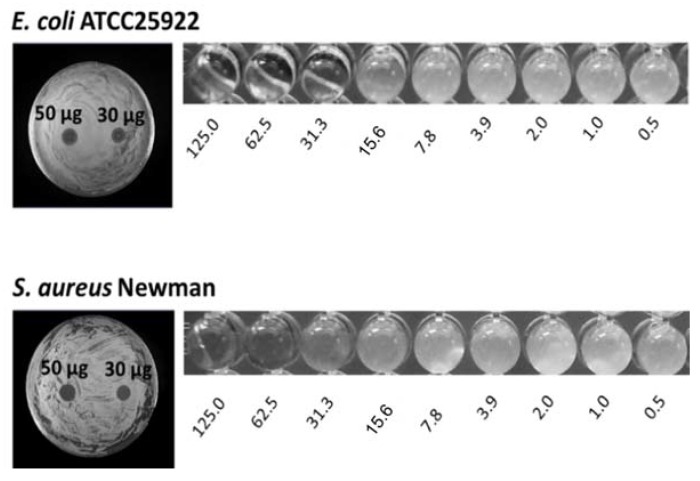
Photographs illustrating results from the assessment of complex **1** antibacterial activity towards *E. coli* ATCC25922 or *S. aureus* Newman by the disk diffusion method and MIC determination by broth microdilution assays. Circular growth inhibition zones are visible in plates spotted with 50 or 30 μg of the complex. The numbers below the wells indicate the respective complex **1** final concentration, in μg/mL. Photographs (not at the same scale) taken after 24 h of incubation at 37 °C.

MIC values of a selection of silver camphor imine complexes towards *E. coli*, *S. aureus*, *P. aeruginosa*, and *B. contaminans* are shown in Table 1. The examples aim to highlight the profound effects of the presence, position, and number of aryl groups in the antibacterial activity of the silver camphor imine complexes. These selected examples also evidence that structural differences in the complexes due to *para* (complex **3**) or *meta* (complex **2**) substituents in the aromatic ring drive significant variations in the MIC values for the strains tested, not only between Gram-positive and Gram-negative strains but also within the Gram-negative strains. From the set of bicamphor complexes in Table 1, [Ag(NO_3_)(OC_10_H_14_N)_2_(*p*-C_6_H_4_)] (**3**, ligand of type A3, Figure 4) displays the highest antibacterial activity against Gram-negative strains, followed by complex **1** with a type A5 ligand (Figure 4). In both cases, electron delocalization throughout the camphor ligand may not be innocent since it affects the electron density at the silver center and consequently the electron transfer processes in which the silver ion may be involved. The MIC values for complexes **1**, **3**, and **4** show that one aromatic group between the two camphor moieties (**3**) increased the antimicrobial activity towards the Gram-negative strains, while two sequential aromatic groups in between the camphor moieties (**4**) resulted in a general loss of antibacterial activity (Table 1). Comparing the biological activity of complexes **2** and **3** (selected to illustrate the effects of the geometry of the ligand in the MIC values according to a relevant effect of geometry and/or electron delocalization on activity), a marked decrease in activity is observed upon replacement of a *para* (**3**) by a *meta* (**2**)-substituted aromatic spacer (Table 1).

A step forward in this work considers the design of new camphor ligands and the synthesis of silver and eventually other metal complexes to investigate the effects of electron density, electron delocalization, distinct geometries, and substituents at the camphor skeleton on the antibacterial activity. The identification of the complexes’ bacterial targets is expected to enable the design of suitable ligands to tailor complexes with enhanced antimicrobial activity and allow rationalization of the mechanisms.

A major concern when developing novel antimicrobials is the emergence of resistant strains. Preliminary unpublished work from our team indicates that the frequency of spontaneous emergence of resistance for *E. coli* ATCC25922, *P. aeruginosa* 477, *B. contaminans* IST408, and *S. aureus* Newman is lower than 4 × 10^−10^. These data were obtained after spreading approximately 10^9^ CFUs of each strain onto the surface of Mueller-Hinton solid medium containing twice the estimated MIC concentration of complex **3** and enumerating the CFUs after five days of incubation at 37 °C. Another issue that still needs to be addressed is the toxicity of the silver camphorimine complexes to humans and animals when envisaging their use in human and veterinary medicine.

## 6. Conclusions

Camphor and silver derivatives have been used since ancient times as medicines. Although their combination in complexes and the search for their antimicrobial activities only started recently, there is no application of silver camphor imine complexes in medicine, pharmacy, or industry. The results obtained until now show that some silver camphor imine complexes combine antimicrobial activity against bacteria and fungi with cytotoxic activity [42,43,44]. This feature is highly relevant since opportunistic bacteria and fungi usually develop during cancer treatments due to the immunosuppressive effects of anticancer drugs.

The insights already made into the antimicrobial properties of some silver camphor imine complexes show they have moderate to high antibacterial activity, depending on the characteristics of the ligands. Such information fosters further enhancement of the ligands to optimize the antibacterial properties of the camphor imine complexes. Meanwhile, synthetic strategies to synthesize, characterize, and evaluate the antimicrobial properties of silver camphor imine complexes were developed and described in the present minireview. Detailed knowledge of the bacterial targets of these compounds is still missing, and therefore, future work will focus on the unveiling of the molecular targets and mechanisms underlying their antimicrobial activity. To address these issues, studies will focus on the use of transcriptomic approaches to gain clues regarding the bacterial gene expression responses to exposure to inhibitory concentrations of silver camphor imine complexes. It will be also necessary to use more classical biochemical approaches to identify the bacterial targets of these complexes. The comprehensive knowledge of the molecular details of the antimicrobial activity of silver camphor imine complexes is expected to enable the exploitation of structure and activity relationships to tailor complexes with enhanced antimicrobial activity.

## Figures and Tables

**Table 1:** Minimal inhibitory concentration (MIC) values calculated for complexes [Ag(NO_3_)L], in μg/mL, towards the bacterial strains *Staphylococcus aureus* Newman, *E scherichia coli* ATCC25922, *Pseudomonas aeruginosa* 477, and *Burkholderia contaminans* IST408. Data taken from previous publications [42,43,44].

Ligand (L)	Complex	*S. aureus*	*E. coli*	*P. aeruginosa*	*B. contaminans*
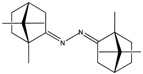	**1**	66 ± 5	50 ± 1	56 ± 4	79 ± 4
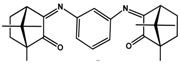	**2**	183 ± 3	65 ± 2	121 ± 2	144 ± 1
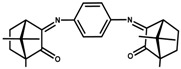	**3**	73 ± 2	20 ± 1	19 ± 4	36 ± 3
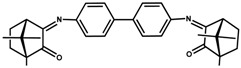	**4**	˃100	˃100	86 ± 7	˃100
AgNO_3_ (Control)	**-**	73	47	39	74
